# Impact of salmon farming in the antibiotic resistance and structure of marine bacterial communities from surface seawater of a northern Patagonian area of Chile

**DOI:** 10.1186/s40659-024-00556-4

**Published:** 2024-11-10

**Authors:** Javiera Ortiz-Severín, Christian Hodar, Camila Stuardo, Constanza Aguado-Norese, Felipe Maza, Mauricio González, Verónica Cambiazo

**Affiliations:** 1 Laboratorio de Bioinformática y Expresión Génica, Instituto de Nutrición y Tecnología de los Alimentos (INTA), Universidad de Chile, El Líbano, 5524 Santiago, Chile; 2 Millennium Institute Center for Genome Regulation (CRG), Santiago, Chile; 3 Laboratorio de Bioinformática y Bioestadística del Genoma, INTA, Universidad de Chile, Santiago, Chile

**Keywords:** Marine bacterial communities, Salmon farms, Metagenomics, Culture enrichment, Antibiotic resistance, Anthropogenic pollution, Environmental risk

## Abstract

**Background:**

Aquaculture and salmon farming can cause environmental problems due to the pollution of the surrounding waters with nutrients, solid wastes and chemicals, such as antibiotics, which are used for disease control in the aquaculture facilities. Increasing antibiotic resistance in human-impacted environments, such as coastal waters with aquaculture activity, is linked to the widespread use of antibiotics, even at sub-lethal concentrations. In Chile, the world's second largest producer of salmon, aquaculture is considered the primary source of antibiotics residues in the coastal waters of northern Patagonia. Here, we evaluated whether the structure and diversity of marine bacterial community, the richness of antibiotic resistance bacteria and the frequency of antibiotic resistance genes increase in communities from the surface seawater of an area with salmon farming activities, in comparison with communities from an area without major anthropogenic disturbance.

**Results:**

The taxonomic structure of bacterial community was significantly different between areas with and without aquaculture production. Growth of the culturable fraction under controlled laboratory conditions showed that, in comparison with the undisturbed area, the bacterial community from salmon farms displayed a higher frequency of colonies resistant to the antibiotics used by the salmon industry. A higher adaptation to antibiotics was revealed by a greater proportion of multi-resistant bacteria isolated from the surface seawater of the salmon farming area. Furthermore, metagenomics data revealed a significant higher abundance of antibiotic resistant genes conferring resistance to 11 antibiotic families in the community from salmon farms, indicating that the proportion of bacteria carrying the resistance determinants was overall higher in salmon farms than in the undisturbed site.

**Conclusions:**

Our results revealed an association between bacterial communities and antibiotic resistance from surface seawater of a coastal area of Chile. Although the total bacterial community may appear comparable between sites, the cultivation technique allowed to expose a higher prevalence of antibiotic resistant bacteria in the salmon farming area. Moreover, we demonstrated that metagenomics (culture-independent) and phenotypic (culture-dependent) methods are complementary to evaluate the bacterial communities’ risk for antibiotic resistance, and that a human—influenced environment (such as salmon farms) can potentiate bacteria to adapt to environmental stresses, such as antibiotics.

**Supplementary Information:**

The online version contains supplementary material available at 10.1186/s40659-024-00556-4.

## Introduction

Aquaculture, defined as the rearing or cultivation of aquatic organisms using techniques designed to increase their production beyond the natural capacity of the environment [1], is one of the pillars of sustainable food supply and its production has increased notably over the years [2]. With a total production of 87.5 million tons of aquatic animals in 2020, mostly for use as human food [3], is considered the primary source of antibiotics in coastal waters [4]. FAO listed 15 countries that lead aquaculture production [3], among them Norway, Chile and China dominate marine farming of fish species. Marine/coastal aquaculture production has been developed mostly by floating cages, and these extensive and semi-extensive systems have been pinpointed as a source of pollution for the surrounding waters, due to the emission of nutrients, solid wastes and chemicals with possible adverse effects for the local aquatic environment [5]. In Chile, aquaculture has grown exponentially since the 80 s, mainly due to increased salmonid rearing. With an annual production between 727 thousand and over a million tons for the last ten years [6], Chile became the second largest producer of farmed salmon in the world. The intensive culture of fish in high densities affects fish physiology and increases their levels of stress [7]; as a consequence, the susceptibility to a variety of infectious diseases also increases [8]. As control of infectious diseases heavily relies on the use of antibiotics, large amounts of antibiotics can be administered to farmed salmonids [9]. In Chile, over 300 tons of antibiotics are used each year by the salmon farming industry via medicated feed [6] and, therefore, not only the organic waste from the farms is dispersed by the ocean, but also the antibiotics [10, 11]. The continuous alteration of the ecological balance in the complex marine system has important implications for the environment and human health, including the loss of biodiversity and the appearance of antibiotic resistant bacteria (ARB) [12–18].

The presence of pollutants from anthropogenic origin in aquatic environments raises serious issues in terms of environmental protection and ecological safety, and antibiotics, a distinctive class of environmental pollutants, have attracted substantial attention due to their widespread presence in aquatic environments around the world [19–21]. Moreover, anthropogenic activities have been connected to the spread of antibiotic resistance genes (ARGs) [22] and ARB [23] in aquatic environments. Previous studies have shown the presence of ARB in sediments below salmon farm cages and in salmon themselves [24, 25], and high levels of antibiotic resistance in bacteria isolated from marine sediments in both aquaculture and non-aquaculture sites has been reported [24]. Since these studies have mainly relied on isolated bacteria, which limits the number and diversity of microorganisms that can be analyzed for the presence of ARGs [26–28], in the present study we used culture-dependent and culture-independent metabarcoding approaches to uncover the structure and diversity of the microbial communities recovered from the surface water in salmon cages within an Aquaculture Farm Site (AFS) and from a undisturbed area at the Pacific Ocean shore (PSh), where direct anthropogenic interference associated to aquaculture activities is minimal to nonexistent.

Currently, there are no standardized methods for antibiotic susceptibility testing of environmental bacteria, and most methods used for investigating environmental ARB consist of obtaining isolated bacteria and testing their resistance to selected antibiotics [29, 30]. However, bacteria form complex networks in the marine environment, and several findings have highlighted the importance of bacterial interactions in antibiotic resistance, as structured bacterial communities can provide antibiotic protection due to antibiotic degradation [31], biofilm formation [32] or horizontal gene transfer [33]. In this work, we investigated the genetic determinants of antibiotic resistance by shotgun sequencing of marine bacterial communities and the phenotypic resistance of the communities by growing the culturable fraction under controlled laboratory conditions. We hypothesized that antibiotic resistance gene (ARG) abundance and phenotypic resistance in bacteria will be increased in sites with higher exposure to the anthropogenic pollution generated by salmon farming activities. In addition, by identifying the members of the bacterial communities of AFS and PSh site we expected to find concomitant changes in bacterial populations associated with an increased genetic and phenotypic antibiotic resistance. Throughout these complementary approaches, we aimed to reveal the bacterial communities’ risk for antibiotic resistance in a human-influenced environment, such as salmon farms, and also to uncover ARB and ARGs in a distant environment to provide a view on background levels of perturbation.

## Materials and methods

### Sample collection and processing

Two sampling sites in southern Chile were chosen: (1) an aquaculture production area in the western side of the Chiloe Island, with high density of salmon farms, named as Aquaculture Farm Site (AFS), hereinafter AFS; (2) a site from the eastern side of the island, near the protected area “Monumento natural Islotes de Puñihuil”, named as Pacific Ocean shore (PSh), hereinafter PSh. The surface seawater samples from AFS and PSh were collected in springtime in areas approximately 30 m deep using sterile opaque plastic containers. The site details (including georeferenced locations) and the water physicochemical parameters are provided in Additional file 1. At both sites, 20 L of surface seawater were collected in different sampling points from each site to create replicates.

For the metagenomics and 16S metabarcoding studies, five replicate filters per site containing the pico-nanoplanktonic fraction (cell sizes > 2 µm) were obtained using a sterile filtration system composed of three filters with different pore sizes (200-µm nylon mesh, 3.0 μm sterile Whatman filter and 0.22 μm Sterivex filter). Filters were preserved in RNA Save (Biological Industries) for transportation and stored at − 80 °C until processing. For the cultured bacterial experiments, two liters of water per site were filtered using the sterile filtration system as mentioned, but the final fraction was collected with 0.22 µm Whatman filters instead of Sterivex filters (0.5 L per filter in 5 replicate filters). The 0.22 µm filters were kept at 4 °C until processing.

### Culture conditions and experimental design

Culturable microbiota from the water samples were obtained from the 0.22 µm Whatman filters by plating in 7 different agar media supplemented with 0.25 µg/mL amphotericin B (Gibco, Thermo Fisher) to prevent fungal growth (see Additional file 1 for detailed composition of media and growing conditions). Plates were incubated at room temperature for up to 7 days to collect the “Culturable community” pool (i.e. the bacterial mixture able to grow in the seven culture media tested). Aliquots from those pools were subjected to DNA purification and used as an inoculum for the subsequent growth experiments. The diluted LB agar (“Nutrient medium”, NM) and R2A agar in seawater (“Saline medium”, SM) were selected for the antibiotic susceptibility assays, considering that they presented the highest bacterial growth and contrasting nutrient composition. Antibiotic susceptibility was tested in both media by plating serial dilutions of the Culturable communities from all the replicates from each site and incubating for 7 days at room temperature. The antibiotics and doses used were: 10 µg/L florfenicol (FFC, Sigma-Aldrich), 10 µg/L flumequine (FLQ, Sigma-Aldrich), or 25 µg/L oxytetracycline (OTC, Sigma-Aldrich). Frequency of resistant colonies was determined (see Additional file 1) and aliquots of bacterial communities grown in NM and SM, with and without antibiotics, were collected for DNA purification and 16S rRNA sequencing.

In addition, isolate collection was created by isolating colonies that grew in the five culture media described in SI, after 5 days of incubation at room temperature. Taxonomic identification was determined by sequencing the 16S rRNA from the isolates, using 91 distinct colonies successfully recovered from the PSh site, and 87 distinct colonies from AFS (isolate collection). Isolates were exposed to antibiotics as described in Additional file 1.

### DNA purification

Sterivex filters were thawed on ice and gently washed with sterile PBS to remove the RNA Save solution. Filters were cut in slices and introduced in sterile 1.5 mL tubes for subsequent DNA extraction for the “Marine bacterial community” samples. In parallel, aliquots with bacterial pellets from the five replicates obtained from the Culturable communities, the communities growing on NM and SM with and without antibiotics, and the isolate collections from each sampling site were also collected for DNA extraction. DNA was purified according to the protocol described by Tillet et al. 2000 [34] with some modifications. The filters and the bacterial pellets were suspended in xanthogenate buffer [1% potassium ethyl xanthogenate (Sigma-Aldrich, United States), 100 mM TrisHCl (pH 7.4), 20 mM EDTA (pH 8), 800 mM ammonium acetate] with 1% SDS. The mixture was incubated at 65 °C for 2 h and placed on ice for 30 min. The DNA was then purified with phenol–chloroform-isoamyl alcohol (25:24:1), followed by chloroform isoamyl alcohol (24:1). The DNA was precipitated overnight with cold isopropanol (− 20 °C) and subsequently washed with 70% ethanol. The DNA quality was evaluated by spectrophotometry (A260/A280 ratio) in a Nanoquant spectrophotometer (Tecan), and the integrity was verified by standard 1% agarose gel electrophoresis. DNA was quantified using a Qubit 2.0 Fluorometer (Thermo Fisher Scientific).

### 16S rRNA gene amplicon sequencing and analysis

For the 16S rRNA sequencing of all samples, the V1–V3 hypervariable region of the 16S rRNA gene was amplified from each purified DNA sample and Illumina MiSeq platform was used to generate the sequencing data. Detailed information on Illumina MiSeq sequencing, processing and diversity analysis of sequencing data are given in Additional file 1. The list of amplicon sequence variants (ASVs) with their associated taxonomy, number of reads and relative abundance is shown in Additional file 2. Differentially abundant taxa were identified by DESeq2 analysis [35], and the results are listed in Additional file 3. The metrics used to evaluate alpha diversity of the bacterial communities were Shannon index and Observed richness, and for beta diversity, Bray‐Curtis dissimilarity.

Individual colonies form the isolate collection were cultivated and their genomic DNA was used as template to amplify the full-length 16S rRNA gene using the universal primers 27F and 1492R. The PCR products were purified and sequenced in Macrogen (Seoul, Korea), and taxonomy was assigned using BLAST (see Additional file 1). The sequences of the 16S rRNA genes were deposited in Genbank under the accession numbers given in Additional file 4.

### Phylogenetic analysis of ASVs and 16S rRNA genes

Phylogenetic analysis was conducted by combining the ASVs obtained from the Marine bacterial communities, the Culturable communities and the communities growing on NM and SM with and without antibiotics, with the full-length 16S RNA gene sequence obtained from the isolate collection. The sequences were aligned using MAFFT v7.508 software [36] and subsequently trimmed the resulting alignment using TrimAI v1.4.rev15, with *automated* parameter [37]. The final tree was inferred using FastTree v2.1, which employs an approximately maximum-likelihood approach [38]. Finally, the figure was constructed by adding annotations using the iTOL v6 web platform.

### Metagenome sequencing, assemblies and annotation

Following quintuplicate DNA extraction from each site, pools were created by combining equal amounts of DNA from each sample, and metagenomic sequencing was performed with an Illumina NovaSeq PE150 platform (see Additional file 1 for detailed library preparation protocols, metagenomics sequencing and analysis). The metagenomes sequence data are deposited in NCBI SRA repository, as part of the PRJNA971915 bioproject, under the accession IDs SRR24758006 and SRR24758005, corresponding to biosamples SAMN35521964 and SAMN35521965 for PSh and AFS, respectively.

### ARG prediction, relative quantification, and abundance

The diversity and abundance of ARGs were annotated with RGI using CARD database [39]. The ARG-like reads were then matched against the database using BLASTX. The reads that met the BLASTX criteria (RGI: 50% sequence identity, 30% coverage) were classified according to the CARD database in Antibiotic families, Antibiotic and Resistance mechanism.

To estimate the relative abundance of genes within each metagenome, the reads were mapped to the metagenomes using *bowtie2* software [40]. The relative abundance of the predicted ARGs was computed as Reads Per Kilobase Million (RPKM). This metric was used to compare the metagenomic abundance of co-occurring ARGs on the same metagenome-assembled contigs. The RPKMs were calculated for each gene using the *pileup* function from BBTools package (BBMap—Bushnell B.—sourceforge.net/projects/bbmap/) and then transformed these values using the following equation:

$${TPM(X}_{i})= [ RPKM({X}_{i}) / \sum RPKM(X)]*{10}^{6}$$

TPM counts for each predicted ARG were used to normalize the abundance and compare between samples. ARG prediction, classification and relative abundance are listed in Additional file 5.

To compare the abundance of each antibiotic family, those families with at least five ARG-containing contigs per metagenome were selected and ARG TPMs were transformed by log_2_. Normality was assessed by either the Shapiro–Wilk or Kolmogorov–Smirnov test, depending on the number of contigs within each family. The resulting *p*-values were adjusted using the Benjamini–Hochberg method with a false discovery rate (FDR) set at 5%. The homogeneity of variances was verified by performing pairwise comparisons within each family between the two locations, using an F-test with a significance level of 5%. Finally, comparisons of pairwise mean abundances were conducted between each antibiotic family in both locations using the two-sample Welch's t-test, with a significance level of 5%. All statistical analyses were performed using R 4.3.0 software [41].

## Results

### Bacterial diversity and community composition

**Fig. 1:**
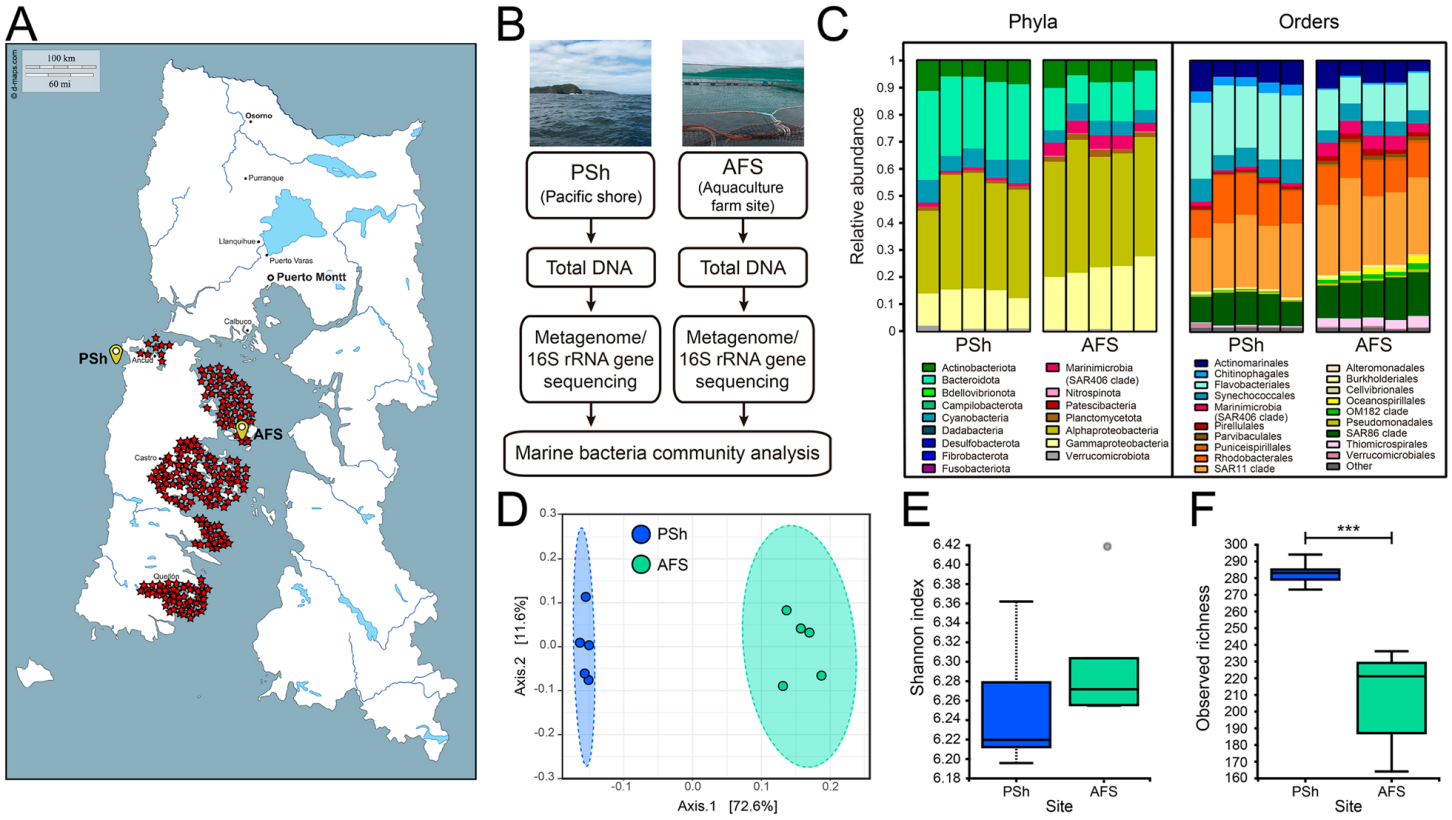
Geographic location, sampling site areas and taxonomic composition of the Marine bacterial communities. **A** Chiloe Island and Los Lagos regional map in northern Patagonia, Chile. The yellow bubbles indicate the sampling sites in PSh and AFS. The red stars indicate aquaculture centers at the time of the sampling. **B** Schematic diagram of the sample processing. **C** Relative abundance graph of the bacterial communities in the PSh and the AFS sites, at the phylum and order taxonomy levels. **D** Beta-diversity analysis. Bray–Curtis with Pairwise PERMANOVA (999 permutations), q = 0.011. **E** Shannon index, Kruskal–Wallis test, H value = 1.32, q = 0.25. **F** Observed richness, Kruskal–Wallis test, H value = 6.82, q = 0.009

To compare bacterial diversity and composition from the two geographical locations studied in this work, the Marine bacterial communities from PSh and AFS were collected and processed as shown in Fig. 1A, B. Amplicon sequencing of the 16S rRNA gene (Additional file 2) identified 326 ASVs across seawater replicate samples from PSh (average = 289 ASVs/sample) and 255 ASVs across AFS replicate samples (average = 208 ASVs/sample). Taxonomic composition at the phylum level (Fig. 1C) highlighted the dominance of Proteobacteria in both samples (> 52%). At the order level, similar relative abundances of SAR11 clade and Rhodobacterales were found at both sites, while higher proportions of Flavobacteriales and Chitinophagales were observed in PSh when compared to AFS. In AFS samples, higher relative abundances of Marinimicrobia (SAR406 clade) and Thiomicrospirales were detected (Fig. 1C). PCoA Bray–Curtis revealed a significant (PERMANOVA q < 0.05) divergence in Marine bacterial communities between PSh and AFS sites (Fig. 1D). The first two principal components accounted for more than 84% of the observed variation in microbiome ASVs. Regarding alpha diversity, Shannon’s index did not differ between sites indicating similar diversity (Kuskall-Wallis q = ns, Fig. 1E), whereas the number of observed ASVs was higher in PSh compared to AFS as indicated by an increased observed richness index in the microbiome from PSh (Kuskall-Wallis q < 0.05, Fig. 1F). A differential abundance analysis was performed to compare the taxonomy from PSh and AFS, finding significant differences (adjusted p-value < 0.05, Additional file 3) in a subset of 83 ASVs comprising six phyla (see Additional file 3).

### Diversity and composition of culturable communities from surface seawater

**Fig. 2:**
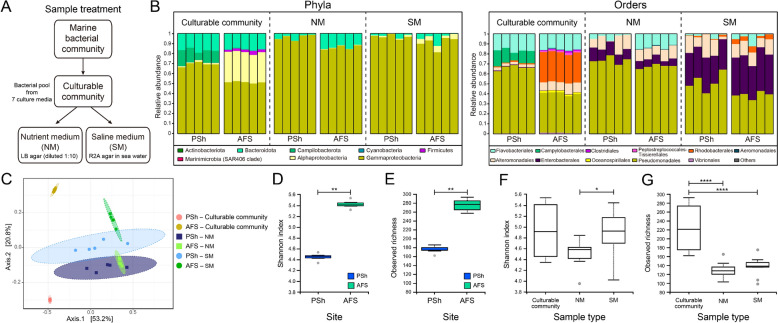
Taxonomic composition of the Culturable communities present in PSh and AFS sites. **A** Schematic diagram of the sample treatment for DNA collection. **B** Relative abundance graph of the Culturable communities in PSh and AFS, at the phylum and order taxonomy level. **C** Beta-diversity PCoA analysis. Bray–Curtis with Pairwise PERMANOVA (999 permutations; pseudo-F = 5.08, q = 0.005). Alpha-diversity analysis comparing the Culturable communities between PSh and AFS: **D** Shannon index, Kruskal–Wallis pairwise test, H value = 14.40, q = 0.0001. **E** observed richness, Kruskal–Wallis pairwise test H value = 3.57, q = 0.058. Alpha-diversity analysis comparing the Culturable communities with those grown on NM and SM in both PSh and AFS. **F** Shannon index (NM vs SM) Kruskal–Wallis pairwise test H value = 6.61, q = 0.030.** G** observed richness Kruskal–Wallis pairwise test (Culturable community vs. NM) H value = 13.73, q = 0.0005. Kruskal–Wallis pairwise test (Culturable community vs. SM) H value = 12.64, q = 0.0005

To assess whether conventional culture-based approaches could be applied to uncover the presence and diversity of antibiotic resistant bacteria, the Marine bacterial communities from PSh and AFS were grown on a battery of culture media and subjected to metabarcoding analysis (Fig. 2A). For PSh and AFS an average of 179 and 278 ASVs were identified, representing seven and six phyla and 21 and 17 orders, respectively (Additional file 2). The composition of Culturable communities (Fig. 2B) indicated that Proteobacteria was the most abundant phylum in the two sites (> 70%), comprised predominantly of Gammaproteobacteria (69.2% in PSh and 51.2% in AFS). At the order level (Fig. 2B), the Culturable communities from PSh and AFS samples were dominated by Pseudomonadales (65.41% in PSh and 39.24% in AFS) and contained similar relative abundances of Flavobacteriales (16.8% in PSh and 15.7% in AFS). Seventy-seven ASVs were detected with significant differences in relative abundance between the PSh and AFS culturable fraction (Additional file 3). Most differentially abundant ASVs in PSh were assigned to Pseudomonadales (19), while in AFS the differentially abundant ASVs mainly comprised Alteromonadales (14), Pseudomonadales (13) and Flavobacteriales (9).

The results of culture enrichment and 16S rRNA gene amplicon sequencing also revealed differences between the Culturable and the Marine bacterial communities, we noticed that 91.4% of the culturable ASVs were not detected by direct sequencing of bacterial communities (N = 362). Moreover, representatives from only 38% of the bacterial orders in the Marine bacterial communities of PSh and AFS were able to grow on the panel of seven culture media. Cultured lineages included 25 orders and 59 bacterial genera, 37 of which were not detected by culture-independent sequencing (Additional file 2).

When the Marine bacterial communities from PSh and AFS were cultured in a Nutrient Medium (NM) or a Saline Medium (SM), the culturable community from either sample or media was dominated by Gammaproteobacteria (> 85% relative abundance). However, differences between the two media were apparent from the relative higher abundance of orders Rhodobacterales, Enterobacterales and Alteromonadales in PSh and AFS communities grown on SM, together with a decrease in Pseudomonadales and Flavobacteriales. Also, most of the under-represented orders (< 2% abundance) from the Culturable communities were lost when grown on the NM and SM. The dissimilarity between the bacteria grown on NM and SM compared to the Culturable community was revealed by Beta-diversity analysis (Fig. 2C). PCoA underlined the separation between the PSh and AFS Culturable communities and also between the PSh and AFS communities recovered from NM and SM, while showing a partial overlapping of the communities from the same site grown on the two different media. Alpha diversity indicated an overall higher diversity and richness in the Culturable community from AFS than in those recovered from PSh (Fig. 2D, E). Considering the three types of Culturable communities, we observed a significantly higher diversity and richness in the communities cultured on seven different substrates as compared to the communities grown on a single medium (Fig. 2F, G), irrespective of the site. Thus, the differences in bacterial richness were directly related to the number of media compositions used (i.e., more culture media, more bacterial richness).

### Changes in the culturable communities exposed to antibiotic-containing media

**Fig. 3:**
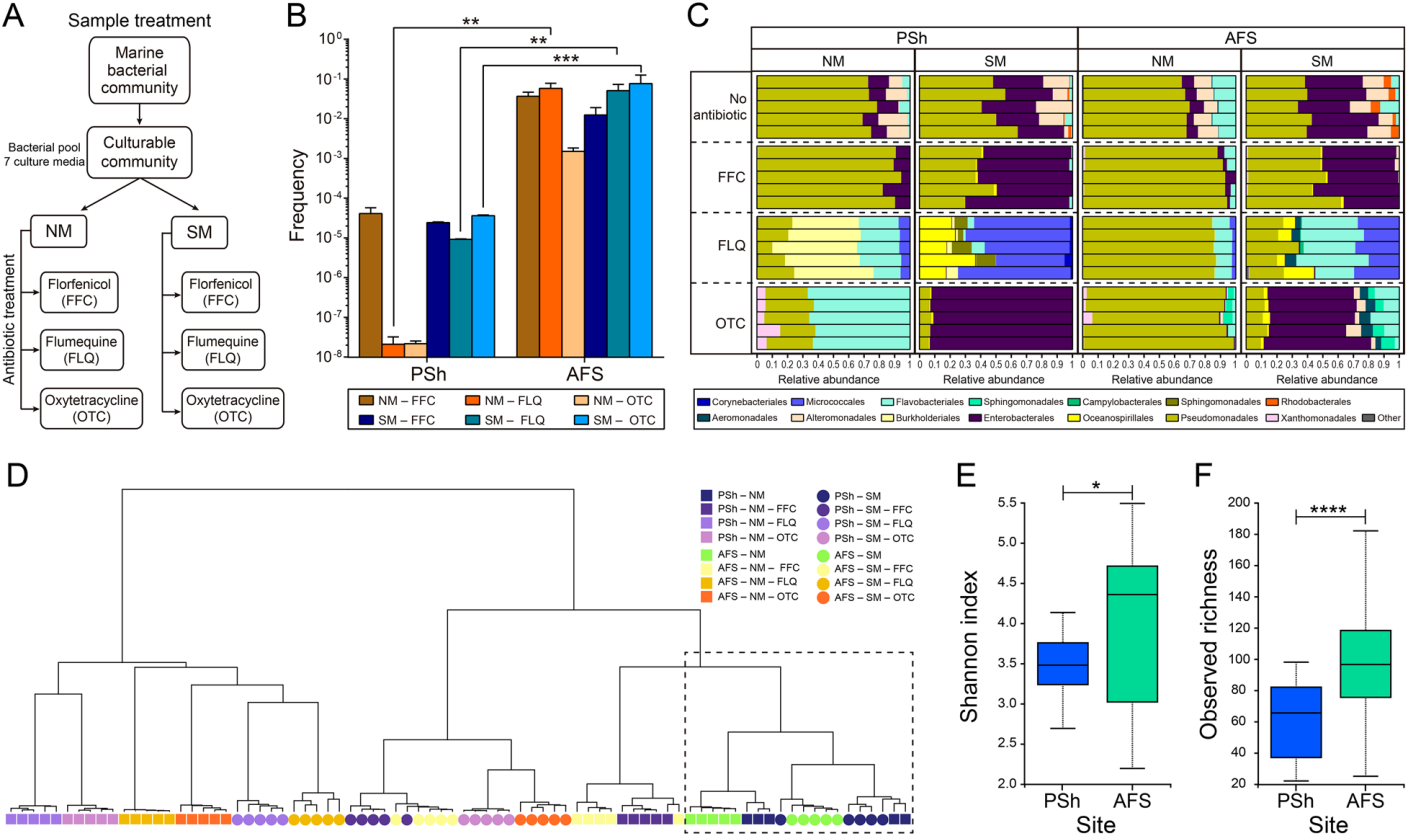
Phenotypic antibiotic resistance and taxonomy of the PSh and AFS Culturable communities. **A** Schematic diagram of the sample treatment, culture media and the antibiotic used prior to DNA collection. **B** Antibiotic resistant colonies recovered in NM and SM plated from the Culturable community. The graphs show the frequency of resistant colonies, as the ratio between the number of resistant colonies and the total number of colonies obtained in a particular medium without antibiotics. **C** Relative abundance graph of the Culturable and antibiotic resistant bacterial communities in the PSh and the AFS sites, at the order taxonomy level. **D** Beta-diversity dissimilarity dendrogram constructed with Ward clustering algorithm. The dashed box shows the samples without antibiotics clustering together. Alpha-diversity analysis comparing the antibiotic resistant ASVs from both media (NM and SM), between the PSh and the AFS sites: **E** Shannon index, Kruskal–Wallis test H value = 6.24, q = 0.01. **F** observed richness, Kruskal–Wallis test H value = 15.42 q = 0.00008

The Culturable bacterial communities from each site were subjected to two culture passages before exposing the bacterial pools to the antibiotics used by the Chilean salmon industry (Fig. 3A and Additional file 1). In the NM, the number of colonies obtained from PSh samples was between 4 and 7 logs less in the antibiotic-containing media than in the control, whereas the number of colonies from AFS was 1 to 2 logs. Similarly, in the SM, a decrease in 4–5 logs in the number of colonies was observed in PSh samples in the antibiotic-containing media compared to the control, and only from 1 to 2 logs in the AFS samples, depending on the antibiotic used. For example, the frequency of FFC (the antibiotic most used by the salmon industry) resistant colonies in NM was 3.68 × 10^–2^ ± 9.6 × 10^–3^ for AFS and 4.11 × 10^–5^ ± 1.6 × 10^–5^ for PSh, whereas in SM the frequencies for AFS and PSh were 1.24 × 10^–2^ ± 6.67 × 10^–3^ and for 2.43 × 10^–5^ ± 1.0159 × 10^–6^, respectively. Consequently, the frequency of resistant colonies was significantly higher in the AFS Culturable community irrespective of the media and the supplemented antibiotic (Fig. 3B).

Multi-resistant bacteria were found in both sites, 269 and 346 ASVs from PSh and AFS, respectively, were resistant to one or more of the tested antibiotics. Of those, 42% in PSh and 51% in AFS were resistant to more than one antibiotic. Specifically, in PSh 20.8% of ASVs were resistant to two antibiotics and 21.2% to three antibiotics, whereas in AFS also 20.8% of ASVs were resistant to two antibiotics but over 30.3% were resistant to three.

Regarding the taxonomic composition, differences were detected among sites, media, and antibiotics (Additional file 2; Fig. 3C). For instance, in PSh the most abundant orders were media and antibiotic-dependent (Pseudomonadales for NM–FFC (88.7%), Burkholderiales for NM–FLQ (49.4%), Flavobacteriales for NM–OTC (64.2%), Enterobacterales for SM–FFC (55.4%) and SM–OTC (92.46%), and Micrococcales for SM–FLQ (60.8%)). On the other hand, in AFS the order Pseudomonadales was the most abundant in NM with the three antibiotics (91.0% for NM–FFC, 85.6% for NM–FLQ and 90.9% for NM–OTC), and in SM–FFC (49.4%). Enterobacterales showed the highest relative abundance in PSh and AFS communities grown on SM–FFC, and four ASVs identified at the genus level as *Serratia* were particularly abundant (50.5% relative abundance in PSh and 40.1% in AFS). On the contrary, Alteromonadales were highly sensitive to antibiotics, decreasing from 11% in NM and 13% in SM in both sites to less than 1% in both media supplemented with FFC, and disappearing in the PSh community growing in NM–FLQ and SM–FLQ.

Differences of taxonomic structure in the antibiotic resistant communities are illustrated by the Beta-diversity dissimilarity analysis (Fig. 3D), as observed by NM and SM samples grouping together in the dendrogram (dashed box) and separate from the ASVs grown on NM–FFC, although they belong to the same cluster. ASV groups from both sites, grown in NM–FLQ, SM–FLQ and NM–OTC, were the most dissimilar. Notably, differences between control and treated samples as well as between the sampled sites, were sometimes due to a single or a few highly abundant ASVs (Additional file 2). For example, one ASV identified as the genus level as *Achromobacter* in PSh and one identified as *Pseudomonas* in AFS accounted for 47.41 and 50.74%, respectively of the total relative abundance of the NM–FLQ-resistant communities. Out of 136 unique ASVs identified in the AFS community grown on NM–OTC, only six ASVs accounted for 89.23% of the total relative abundance, and thus the majority of the OTC-resistant community was present at low abundances (< 2% each). The four predominant ASVs in AFS were identified as members of the *Pseudomonas* genus, whereas in PSh community over 66% of the 26 ASVs identified in NM–OTC were affiliated to *Flavobacterium* and 23.5% to *Psychrobacter*. No common ASVs were identified between the AFS and PSh communities recovered from NM–OTC. Consistently, both Shannon and richness indexes were higher in AFS compared to PSh (Fig. 3E, F).

### Profiling resistance phenotypes of bacterial isolates

**Fig. 4:**
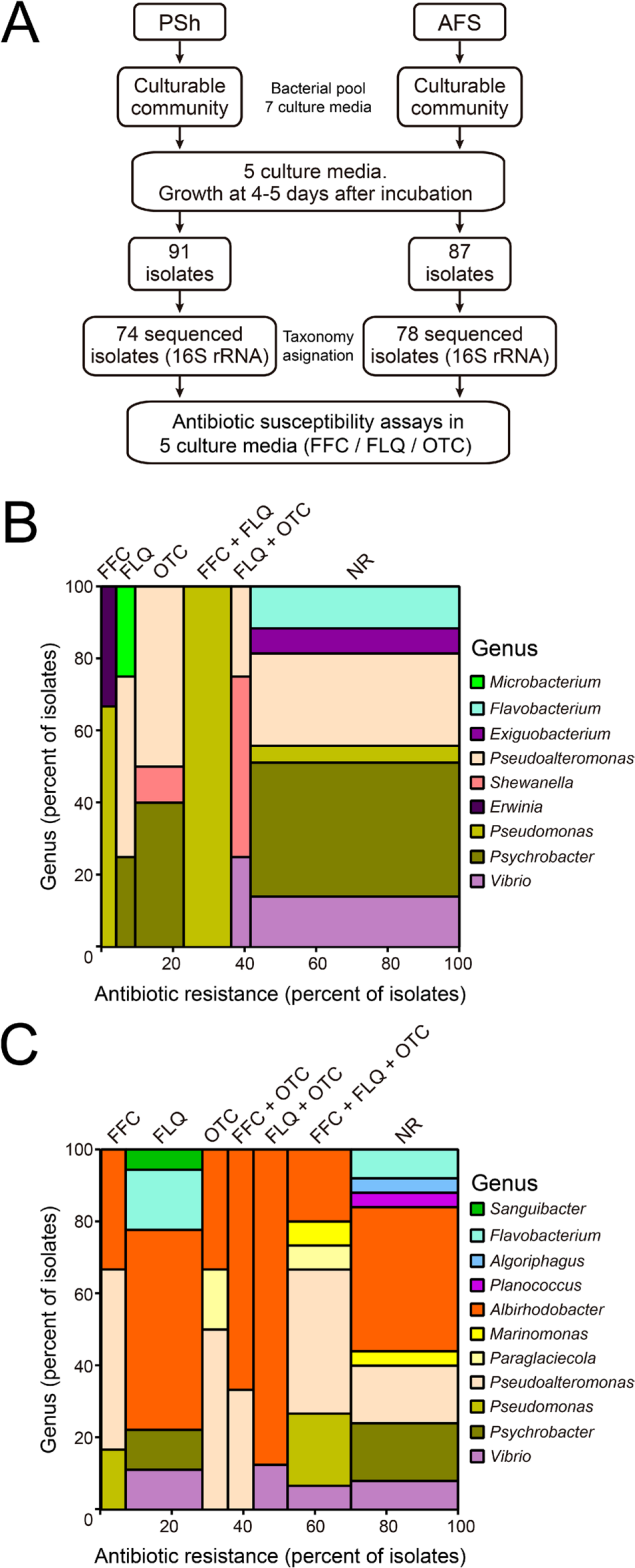
Antibiotic resistance profiles of the PSh and AFS isolates. **A** Schematic diagram of the sample treatment, culture media and the antibiotic used. Isolates were grown in culture media with and without antibiotics to evaluate their antibiotic sensitivity profiles. **B** and **C** mosaic graph showing the identity of the isolates at the genus level (y-axis), related to the proportion of isolates resistant to one antibiotic (FFC, FLQ or OTC), a combination of antibiotics (FFC + FLQ, FFC + OTC, FLQ + OTC, or FFC + FLQ + OTC), or susceptible to all three antibiotics tested (non-resistant, NR). **B** isolates from PSh. **C** isolates from AFS

In order to identify whether antibiotic susceptibility depends on growing as isolates or as members of a community, a total of 152 bacterial isolates were exposed to the antibiotics used by the Chilean salmon industry and their relative susceptibility was evaluated (Additional file 4, Fig. 4A). Bacterial isolates belonged to four phyla (Actinobacteria, Bacteroidota, Firmicutes and Proteobacteria) and included mainly gram-negative species (Additional file 4). Overall, 56.75% of PSh isolates were sensitive to all antibiotics, in contrast with only 21.79% of AFS isolates. Taxonomic analysis of the 16S rRNA sequences showed that bacteria from genus *Psychrobacter* were more abundant in PSh (28.4%) than in AFS (8.11%) and contained the lowest number of resistant isolates (Additional file 4). In PSh, bacterial isolates belonging to *Pseudoalteromonas* were non-resistant or resistant to only one of the antibiotics, although one *Pseudoalteromonas* isolate showed resistance to both FFC and FLQ. In contrast, 12 out of 14 representatives of *Pseudomonas* were resistant to any of the three antibiotics tested. *Pseudomonas* isolates from AFS were also resistant to the antibiotics tested. Only one member of order Micrococcales (genus *Microbacterium*) was isolated from PSh and was resistant to FLQ, and the only member of *Erwinia* genus was resistant to FFC, whereas all *Flavobacterium* and *Exiguobacterium* members isolated from PSh were unable to grow in any of the antibiotics tested (Fig. 4B). On the other hand, five *Flavobacterium* members were isolated from AFS, three of them resistant to FLQ (including two *Flavobacterium piscis*). The only member of Micrococcales order in AFS was *Sanguibacter*, and similarly to PSh, it was resistant only to FLQ. Isolates resistant to FFC in PSh belonged to two genera (*Erwinia* and *Pseudomonas*), whereas in AFS *Albirhodobacter*, *Paraglaciecola*, *Pseudoalteromonas*, *Marinomonas*, *Pseudomonas* and *Vibrio* isolates were resistant to this antibiotic. Also, in AFS the most abundant genus was *Albirhodobacter* (order Rhodobacterales) and members of this genus were found to be non-resistant, resistant to one, two or three antibiotics, showing a high plasticity in terms of antibiotic resistance, a similar result was obtained with *Pseudoalteromonas* isolates. In addition, only isolates from AFS were able to grow in the three antibiotics (Fig. 4C, FFC, FLQ and OTC bar).

**Fig. 5:**
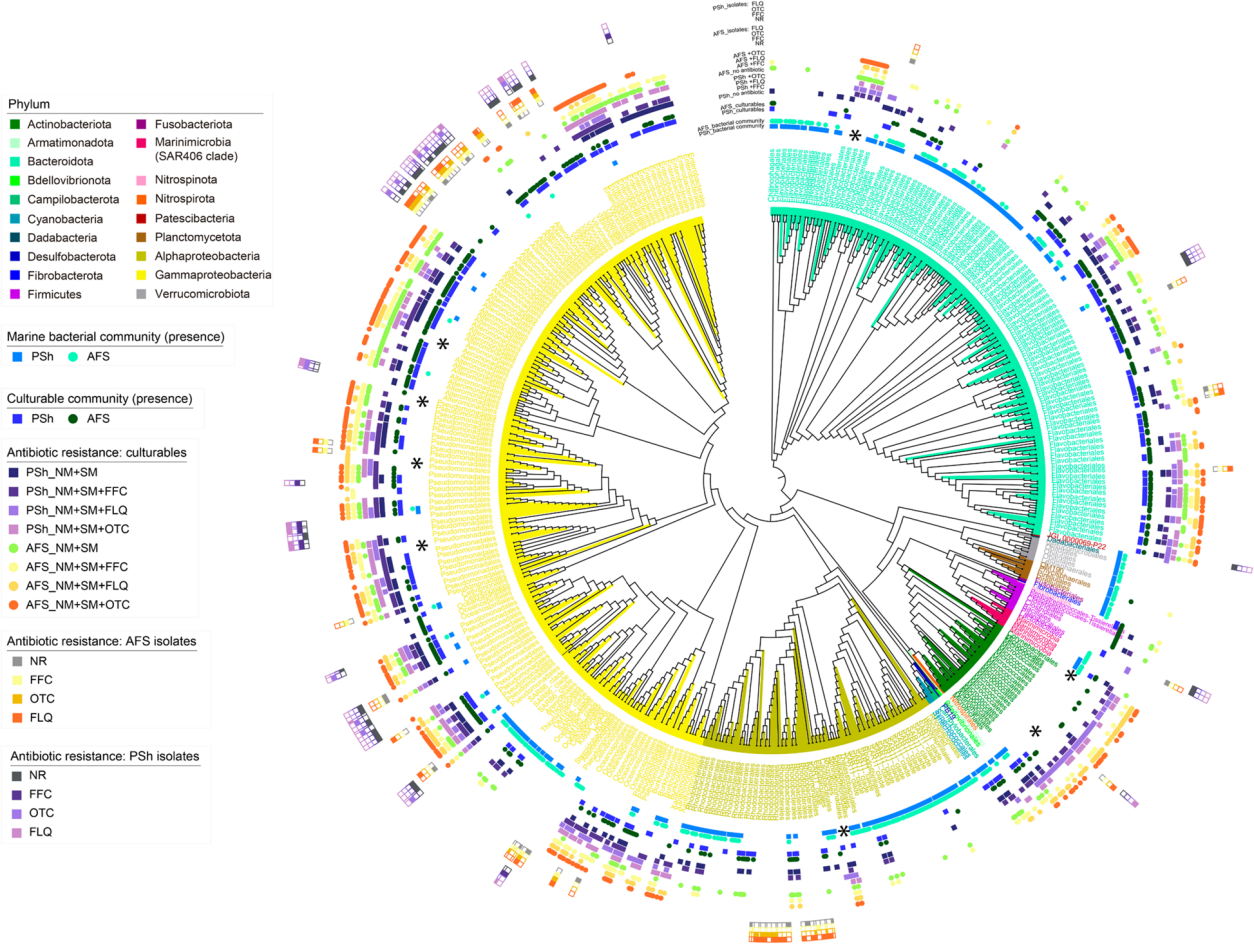
Circular representation of profiled bacterial taxonomies according to the presence of the ASVs in the samples. Maximum likelihood phylogenetic trees of the complete set of bacteria recovered in this study. From the center to the borders, the tree shows ASVs of the Marine bacteria, the Culturable bacteria, from bacteria grown in NM with and without antibiotics, and bacteria grown in SM with and without antibiotics, from PSh (squares) and AFS (circles). The resistance profile of bacterial isolates is shown by filled squares in light yellow, yellow and orange for AFS, and purple, light purple and pink for PSh. The color of the clades corresponds to the phylum taxonomic level, and the ASVs orders are shown in colored letters in the first inner circle. Asterisks indicate taxa recovered after antibiotic exposure

In summary, sequencing results of the Marine bacterial communities (Fig. 1), the Culturable communities (Figs. 2 and 3), the antibiotic resistant fraction (Fig. 3), or the antibiotic-resistant or -sensitive isolates (Fig. 4), revealed that the most represented group is the Gammaproteobacteria, followed by the phylum Bacteroidota (particularly the Flavobacteriales order). Most of the Gammaproteobacteria were preferentially recovered as part of the Culturable communities and belonged predominantly to Enterobacterales, Vibrionales, Alteromonadales and Pseudomonadales orders showing resistance to the different antibiotics tested (Fig. 5). On the other hand, the Flavobacteriales group was identified both in the Marine bacterial and the Culturable communities, showing diverse degrees of antibiotic resistance (Fig. 5). In contrast, ASVs representatives of phyla Verrumicrobiota, Planctomycetota, Fibrobacteriota, Fusobacteriota and Marinimicrobia were mostly detected in the Marine bacterial communities and were unable to grow in cultured media, or independently as isolates (see Fig. 5). In the case of the order Synechoccocales (phylum Cyanobacteria), representatives were found only in the Marine bacterial community and the Culturable community but were unable to grow on NM or SM, or as isolates. Interestingly, some bacterial isolates, particularly from the orders Pseudomonadales and Rhodobacterales, lacked representation in the culturable fractions or the Marine bacterial communities. Thus, the Culturable community from PSh and AFS were composed of bacteria with the capacity to grow in isolation and those who could be recovered only as part of a mixed culture. In some cases, ASVs that were not detected in the Culturable bacteria could be recovered after exposure to antibiotics. For example, members of phyla Bacteroidota, order Sphingobacteriales, Actinobacteriota order Micrococcales and for class Gammaproteobacteria, order Pseudomonadales and Aeromonadales (Fig. 5, asterisks).

### Marine bacterial community and occurrence of ARGs in metagenomes

**Fig. 6:**
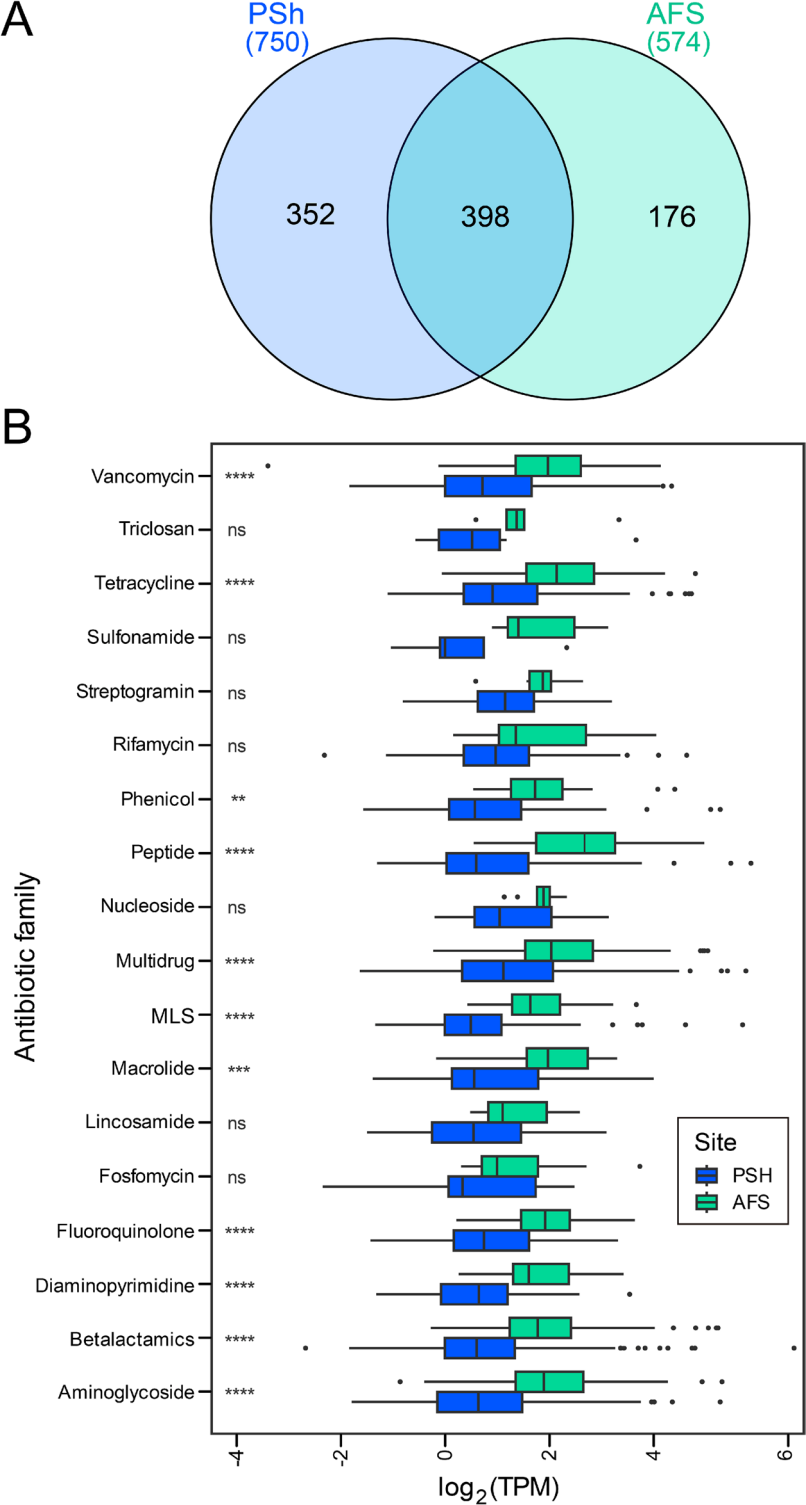
ARG prediction and taxonomy composition of the bacterial communities present in the PSh and AFS metagenomes. **A** Venn diagram of the total number of genes predicted as ARG in both samples. **B** Log_2_ TPM of ARG grouped in antibiotic families. Asterisks indicate significance level (**p < 0.01, ***p < 0.001, ****p < 0.0001, ns non-significate, Welch test)

Shotgun metagenomic data from the surface seawater of PSh and AFS was used to uncover the genetic determinants behind antibiotic resistance in the Marine bacterial communities. As a result of metagenome sequencing and assembly, 205,330 and 165,412 contigs were obtained for PSh and AFS, respectively, with a total of 380,759 and 248,417 predicted proteins for each site. A total of 1,322 genes classified as an ARG with over 60% of sequence identity and 50% of coverage were identified in the PSh contigs, whereas 855 ARGs were predicted in AFS contigs. Of them, 750 and 574 unique ARGs were predicted in the PSh and AFS contigs, respectively, and 398 of them were common to both metagenomes (Fig. 6A).

The ARG abundance calculation based on TPM (Table 1 and Additional file 5) showed that the total abundance of ARGs in PSh was 4,223.2 TPM, almost doubling the abundance of ARGs in the AFS site (2,273.2 TPM). In PSh, ARGs with predicted antibiotic inactivation mechanism had the highest abundance (TPM = 1,956.9), followed by antibiotic efflux, target alteration and target protection mechanisms. In the AFS site, antibiotic efflux together with target alteration were the most abundant resistance mechanisms (Table 1).

**Table 1:** Relative gene abundance (TPM) of predicted ARGs in PSh and AFS metagenomes and classified by resistance mechanism

Resistance mechanism	PSh (TPM)	AFS (TPM)
Antibiotic inactivation	1,956.9	230.72
Antibiotic efflux	937.12	798.41
Antibiotic target alteration	780.43	710.42
Antibiotic target protection	422.21	362.74
Antibiotic target replacement	102.85	139.37
Antibiotic target alteration; antibiotic efflux	10.434	5.2429
Antibiotic efflux; reduced permeability to antibiotic	5.6764	18.878
Reduced permeability to antibiotic	5.5788	7.3988
Antibiotic target alteration; antibiotic target replacement	1.8657	0
Total	4223.2	2273.2

ARG types were matched with their corresponding antibiotic targets and the average relative abundance of the genes conferring antibiotic resistance in the two sites was categorized. The results showed a broad spectrum of ARGs conferring resistance to 25 antibiotic families, with 22 of them shared between the samples. Half of those shared families (11 out of 22 families) were found to have a significantly higher abundance in AFS compared to PSh (Fig. 6B). On the other hand, ARGs conferring resistance to nitroimidazole antibiotics were only identified in PSh, whereas ARGs of the acridine dye, bicyclomycin and mupirocin families were only present in AFS (Additional file 5). In this work, any ARG conferring resistance to three or more antibiotics from different families was categorized as multidrug family. Interestingly, multidrug family was more abundant in AFS (Fig. 6B) revealing a potentially broader spectrum of antibiotic resistance.

ARGs conferring resistance to antibiotics used by the Chilean salmon industry (FFC, FLQ and OTC) were further inspected and grouped in phenicol, quinolone, tetracycline and multidrug families. Although in AFS there was no evident increase in individual gene counts, for these drug families, the abundances (log_2_TPM, Fig. 6B) were significantly higher in AFS than in PSh. Thus, the mean TPM of genes conferring resistant to a given antibiotic family was always higher for AFS than for PSh, for instance, mean TPMs for fluoroquinolone resistance genes were 4.33 in AFS vs. 2.52 in PSh metagenomes, phenicol 4.80 vs. 3.80, tetracycline 5.83 vs. 3.89, and multidrug 5.87 vs. 4.19. Resistance to these antibiotics in PSh occurred predominantly via the action of drug transporters (antibiotic efflux resistance mechanism), while the second and third most predominant antibiotic resistance mechanisms were antibiotic inactivation (mainly chloramphenicol acetyltransferase), and antibiotic target protection, mainly ATP-binding cassette F proteins that confer resistance through ribosome protection (Additional file 5). According to CARD database description, more than 36% of the ARG families conferring resistance to FFC, FLQ and OTC in PSh have been found in mobile genetic elements (MGE, i.e. plasmids, integrons and/or transposons, Additional file 5). Similarly, in AFS, antibiotic efflux was a major resistance mechanism for quinolone, phenicol and tetracycline antibiotics; 48.5% of efflux pump genes from these families were described in CARD database as located in MGE (Additional file 5). The second most predominant antibiotic resistance mechanisms in AFS was antibiotic target protection, mainly quinolone (quinolone resistance protein (*qnr*) and tetracycline resistance (tetracycline-resistant ribosomal protection protein), of which, 40% was detected in MGE.

## Discussion

The Chilean Patagonia has complex topography and hydrographic conditions that influence water exchange between coastal regions and the open ocean, creating micro-environments with unique oceanographic conditions that could be sensitive to excess nutrient input, chemicals and other stressors [42]. Most previous studies on the impact of aquaculture on the environment have been focused on sediments and associated bacteria after isolation [24, 25, 43]. The surface seawater environment of coastal Chilean Patagonia has been less studied, and despite the important perturbations of the marine microbiome as a result of anthropogenic pollution [44, 45], the information regarding the response of associated bacterial communities to aquaculture is limited.

Here, the AFS was considered under high anthropogenic impact, mainly from the presence of more than 300 aquaculture concessions in the area where seawater samples were collected. On the other hand, PSh sample collection site is over 45 km distant from the nearest fish farm and with no aquaculture activities; however, a certain level of perturbation was expected since this site can be exposed to discharges from marine shipping, tourist activities and river inputs.

It is important to note that although we did not conduct antibiotic measurements in the seawater samples, previous work in a nearby area (the Puyuhuapi fjord in Chilean Patagonia) from Jara et al*.* reported the presence of FFC traces 6 months after treatment administration in salmon farms present in the fjord. FFC is by far the most used antibiotic in the Chilean salmon industry, with 118.7 tons of FFC administered where the samples in this work were taken (Los Lagos Region, 2019) [46]. Previously, FFC had been detected in the particulate matter fraction in surface seawater between 7.4 ± 0.9 and 23.1 ± 2.5 ng/L, concentrations several orders of magnitude below the reported inhibitory concentration for the salmon pathogen *Piscirickettsia salmonis* or the laboratory strain *Escherichia coli* ATCC 25922 [47]. The work by Jara’s group suggested that surface sediments with traces of FFC might act as a reservoir for antibiotic resistance bacteria, which is concordant with previous work from Gullberg et al. showing that de novo resistant mutants can be selected at sub-inhibitory antibiotic concentrations and that concentrations up to several 100-fold below the inhibitory concentration of susceptible bacteria can enrich for resistant bacteria even when present at in low initial frequencies[48].

### Bacterial taxonomic composition in surface seawater from PSh and AFS

The Marine bacterial communities of PSh and AFS showed similar trends with regards to the most abundant phyla (Proteobacteria and Bacteroidetes) and orders (SAR11, SAR86, Rhodobacterales and Flavobacteriales), which was consistent with the fact that they are major constituents in several marine systems [49–52]. When comparing the differential abundance of ASVs between PSh and AFS sites, we found that ASVs enriched in the PSh site mainly belonged to the abundant orders, Flavobacteriales, Rhodobacterales and SAR11. In AFS, enriched ASVs belonged to orders Pirellulales, Thiomicrospirales and Marinimicrobia, whose members inhabit low-oxygen habitats [53–56], suggesting the development of hypoxic areas probably due to the eutrophication caused by nutrient enrichment from intensified aquaculture [57, 58]. Marinimicrobia has been also found to be positively correlated with the tetracycline efflux protein, *tetZ* [59]. In addition, facultative hydrocarbon degraders of orders Parvibaculales [60], Kordiimonadales [61–63] and Oceanospirillales were enriched or exclusively found in AFS. Thus, the composition of bacterial communities from AFS suggests the presence of organic pollutants, such as petroleum hydrocarbons, which are known to accumulate in sediments and the surface microlayer [64], and also adsorb to floating plastics [65]. Water pollution from aquaculture facilities, including fish feed, feces, disinfectants and antibiotics affect water quality by increasing the turbidity, decreasing dissolved oxygen levels, and elevating nutrient concentrations leading to eutrophication and harmful algal blooms. Intensive fish and crustacean mariculture are responsible for releasing dissolved and particulate nutrients, causing nutrient loads in coastal areas to escalate. Several studies investigating feed and fish excreta in aquaculture facilities have shown that N and P retention varies between 10 and 49% (N) and 17% to 40% (P) on average, depending on the fish species [66]. Although we did not directly measure pollutants in the surface seawater, shifts in bacterial composition at AFS are concordant with the effects of elevated nutrient levels.

In agreement with the reported taxa of 817 bacterial isolates from photic layers of the North West Mediterranean Sea, the North and South Atlantic Oceans, the Indian, the Pacific, and the Arctic Oceans [67], the culturable fractions of PSh and AFS were highly dominated by Alphaproteobacteria and Gammaproteobacteria. More than 50% of ASVs were affiliated to the genera *Psychrobacter* (order Pseudomonadales) in PSh, and the genera *Albirhodobacter* (order Rhodobacterales) and *Psychrobacter* in AFS. Members of the genus *Albirhodobacter* have been isolated from seashore water or estuary sediment [68, 69], while *Psychrobacter* species have been isolated from marine environments including seawater and marine sediment [70, 71], as part of the normal microbiota of fish skin and sponges and algae tissues [72]. A high proportion of the bacteria isolated from PSh belong to taxa associated with the normal environmental microbiota and shellfish or alga microbiota, while in AFS some of the isolates corresponded to potential fish pathogens from the *Flavobacterium* genus (*F. branchiicola* and *F. piscis*) or were previously recovered from pollute places (*Planococcus ruber*). In addition, ASVs assigned to genus *Aeromonas* were more abundant or exclusively found in AFS and were also resistant to antibiotics FLQ and OTC. Interestingly, two strains of *F. piscis* from AFS, which had been previously isolated from farmed rainbow trout [73] were resistant to FLQ, while most *Albirhodobacter* isolates in AFS were resistant to FFC, constituting a potential source of resistance spreading in the community.

### Culture enrichment as a resource for phenotypic analysis of environmental communities

It is well known that most microbial species including environmental microbiota cannot be isolated due to the limitation of culturing conditions or their inherent characteristics, such as slow growth [74]. Culture enrichment, on the other hand, can be carried out under laboratory to improve the recovery rate of previously uncultured bacteria [75]. Thus, it has been implemented to culture and identify unknown and potentially pathogenic bacteria that inhabit the human gut [76], to describe rare bacterial genera from vertebrate gut microbiota [77], or to carry out phenotypic screening to uncover antimicrobial resistance in wastewater [78]. By integrating culture enrichment and 16S rRNA gene amplicon sequencing, we showed that the compositions of the culturable fractions differed greatly from the environmental samples, hence ASVs not detected in the environmental samples were observed on cultured samples with or without antibiotics. In fact, several ASVs affiliated to the orders Aeromonadales, Flavobacteriales and Micrococcales and to different orders within the Gamaproteobacteria division were not recovered in the Marine bacterial communities (see Fig. 5). Moreover, ASV composition was also distinct between the two sites, indicating that culturing bacteria did not erase the differences observed between the two environmental samples.

Previous works have shown that mixed cultures of environmental bacteria allow the growth of fastidious or difficult-to-grow bacteria, since they can provide growth-promoting factors and enabling bacterial growth under unfavorable conditions [79, 80]. Here, by culturing bacteria as a consortium rather than isolates, we were able to recover bacterial taxa that normally do not grow under standard culture conditions, among them, one member of phylum Cyanobacteria, *Synechococcus CC9902,* which was able to grow as a consortium with the marine Culturable community even in culture media routinely used for heterotrophic bacteria under standard culture conditions. Interestingly, members of yet non-cultivated or poorly cultivated lineages (Clade Ia, Marinimicrobia, NS4 and NS5 marine group, SAR86 clade and SUP05 cluster) were also detected within the culturable fractions of PSh and AFS, although with low abundance. These results suggest that, with our culture approach, we were able to recover a high diversity of culturable bacteria, providing opportunities for further phenotypic assays.

### Bacterial adaptability was revealed by culturing and antibiotic susceptibility assays

After recovering the culturable bacterial fraction from the sediments of an aquaculture site and a control site 8 km away with no observed aquaculture activities, Buschmann et al. [43], reported significant increases in the number of bacteria resistant to oxytetracycline, oxalinic acid and florfenicol in the sediments of the aquaculture site compared to the control site. Moreover, differences in the numbers of resistant bacteria persisted for distances up to 1 km from the aquaculture site, suggesting a long-range effect of antibiotics supplied in salmon farms. On the other hand, Shah et al. [25], using 124 strains isolated from marine sediments at a salmon aquaculture site and 76 strains isolated from a distant control site, reported no significant differences in the levels of resistance of the isolated strains to various antibiotics. The authors attributed this lack of differences in resistance between aquaculture and non-aquaculture sites to the greater amount of antimicrobials used in aquaculture in Chile and their diffusion from the salmon farms. Here, using Culturable communities, we observed a marked difference in antibiotic resistance between the AFS and the PSh. The frequency of resistant colonies was significantly higher in the AFS Culturable community regardless of the media and the antibiotic used in this study. We hypothesize that growing bacteria in a community increases the probability of antibiotic resistant bacteria to proliferate as well as bacteria that can exert a protective effect on more susceptible strains. The ASVs affiliated to the order Micrococcales were exclusively recovered in the antibiotic-supplemented culture media, the same was observed for some ASVs of orders Sphingobacteriales and Pseudomonadales and Aeromonadles, suggesting that their growth was favored when the interaction patterns among community members were modified. It has been reported that interspecies interactions can affect the outcome of antibiotic treatments [81, 82]. Thus, in addition to the modification in the nutritional matrix provided by the culture media, competitive and cooperative relationships among bacteria need to be considered to understand the assembly of microbial communities from enriched cultures.

A variety of ASVs in the culturable fraction were found to be resistant to two or three antibiotics. In both sites, multi-resistant ASVs belonged to phyla Actinobacteria, Bacteroidota and Proteobacteria were detected. In previous works, isolates from genus *Pseudomonas* were commonly found when searching for antibiotic resistant bacteria in salmon farms and salmon-related environments [83–85], and in mariculture systems, Proteobacteria and Bacteroidetes had the higher potential to carry ARGs [14]. We found that several genera from Proteobacteria phylum were multi-resistant, predominately *Pseudomonas* (the genera with more ASVs) in both PSh and AFS, and resistant *Psychrobacter* members were identified only in AFS or with increased abundance compared to PSh. In addition, multi-resistant ASVs assigned to *Aeromonas* and *Flavobacterium* genera were also identified only (or with higher abundance) in AFS, both of which are among bacterial fish pathogens.

According to our results, bacterial diversity was not decreased in the Marine bacterial communities associated with salmon farming (AFS) when compared to PSh, as suggested previously for aquatic bacteria in sediments [86]. Moreover, the culturable fraction of the AFS community, with or without antibiotics, showed an increased diversity when compared to the PSh community. This suggests plasticity in the bacterial response to new environments (i.e. laboratory conditions and culture) and stresses (antibiotics) and also places the culture enrichment approach as a well-suited technique to uncover unexpected features of the bacterial communities.

### Metagenomics revealed differences in potential antibiotic resistance between PSh and AFS

Antibiotic resistance is regarded as a critical “One Health” challenge that has been linked to humans, animals, and environmental factors [87]. The processes of emergence and spread of antibiotic resistance genes (ARGs) on the microbiota of humans and animals and also in various environments [88] are of particular concern, and require an effective surveillance of ARGs in natural environments, especially those associated with anthropogenic activities. Several lines of evidence support the hypothesis that increasing antibiotic resistance in human-influenced environments is linked to the widespread use of antibiotics, even at sub-lethal concentrations [14, 32, 89–92].

Large-scale studies have reported a higher abundance of ARGs conferring resistance to quinolone, bacitracin [93, 94] and betalactamics [95] in coastal waters, where they seem to strongly correlate with the anthropogenic influences on coastal marine ecosystems [23, 96]. Other works evaluating bacterial resistome have observed an increased incidence of ARGs in environmental samples that were subjected to antibiotic pressure [97, 98]. Here, an increased antibiotic resistance potential was revealed in the AFS site only when the abundance was determined in ARGs grouped by Antibiotic families, since the number and abundance of individual ARGs were higher in PSh. However, half of the Antibiotic families present in both sites were significantly more abundant in AFS than in PSh, including those conferring resistance to the studied antibiotics (tetracycline, fluoroquiniolone and phenicol) and those conferring muti-resistance by the multidrug Antibiotic family. In this sense, antibiotic efflux, which was highly abundant in AFS, is considered as the main mechanism responsible for the development of simultaneous resistance to multiple antibiotics [99], which is concordant with the higher proportion of multi-resistant isolates found in AFS compared with PSh isolates. Also, the resistance mechanisms of the antibiotics used by the salmon industry were more diverse in AFS than in PSh, which also correlates with the increased abundance of antibiotic resistant bacteria in AFS.

Since ARGs are often spread among bacteria by horizontal gene transfer facilitated by MGE [100] we used the CARD database to show that close to 40% of the main ARGs families conferring resistance to quinolone, phenicol and tetracycline antibiotics, in both PSh and AFS, were located in MGE. The importance of MGE in the dynamics of ARG transmission have been reported in several natural environments, including a recent global analysis of 293 metagenomics samples from the TARA Oceans project [93], indicating that 24.76% of the predicted ARGs were present in contigs classified as plasmids. Further analysis will be needed to thoroughly describe the mobile part of the metagenomes reported in this study and predict their horizontal dispersal.

We observed an increased abundance in a variety of ARG grouped in antibiotic families, not only in those associated with the antibiotics used by the salmon industry, which suggests that the anthropogenic pollution created by salmon farming activities creates an environmental pressure conducive to unspecific or undirected antimicrobial resistance, which can be observed both by metagenomics (culture-independent) and phenotypic (culture-dependent) methods. At this point, however, it is not clear whether the antibiotic treatment administered to the fish over long periods (since the 90’s in the Chilean salmon industry), or other environmental perturbations (such as an increase in nutrient availability via salmon feed or salmon waste, the boat’s oil or fuel, etc.) or both that cause these changes in the bacterial ARGs repertoire that increases their antibiotic resistance potential. Nevertheless, it is known that the selective pressure exerted by antibiotics can be a main factor in the development of resistance. For instance, the use of broad-spectrum antibiotics in aquaculture can lead to the selection of multidrug-resistant bacterial strains [85, 101]. Moreover, the selective pressure for antimicrobial resistance is not limited to antibiotics since other compounds such as heavy metals or pesticides can also play a role in in the development of resistance [102]. For example, the presence of copper and zinc, has been associated with the co-selection of antibiotic resistant strains in different environments [103]. These ARGs can then disseminate to other bacteria through vertical- and horizontal gene transfer [104, 105].

Although our comparative analysis of ARGs and ARBs present in AFS and PSh demonstrates the value of having reference samples taken away from aquaculture disturbances, an important limitation of this research is that our data were composed of a single sampling campaign, therefore, we are unaware of how seasonal variations affect the abundance and composition of ARGs and ARBs at the sites under study. In addition, our sampling strategy was geographically restricted, so we cannot confirm our results by comparison with other coastal areas both pristine and intervened by aquaculture activities. Further studies are needed to investigate the prevalence and co-occurrence of ARB, ARGs, antibiotics and various associated chemical compounds in aquaculture and control sites along coastal waters. Certainly, multifaceted approaches are necessary to address the complexity and variability of antibiotic resistance patterns and to provide a comprehensive assessment of antibiotic resistance risks in Chilean coastal waters.

## Conclusions

The comparative study of marine bacterial communities from a site with high anthropogenic impact (salmon farms) and a distant, undisturbed site, revealed that bacterial communities have similar diversity and share major taxonomic groups between sites. However, after culture and antibiotic exposure, an increased diversity of bacterial communities from the salmon farming site was exposed. The composition and abundance of ASVs not only confirmed that the culturable fraction of marine bacteria differs from the environmental samples, but also showed that they can be distinguished based on their alpha and beta diversity metrics. Our cultured-based approaches allowed to recover under-represented and difficult-to-grow taxa, and to identify interesting groups such as pathogens, multi-resistant bacteria, and bacteria that thrives in low-oxygen and polluted environments, proving the complementary nature of the culture-independent to the culture-dependent methods. The response of the culturable communities and bacterial isolates to antibiotic exposure revealed different capacities for antibiotic resistance, which were associated with the origin of the environmental samples. Since a higher abundance of ARGs conferring resistance to 11 antibiotic families was detected in the bacterial communities from salmon farms, our metagenomic data suggested that this phenotypic trait may be the result of the observed difference in the repertoire of ARGs, and the proportion of bacteria harboring them, between the two communities of marine bacteria.

## Supplementary Information

Additional file 1: Methods S1. Extended sample collection protocol, methodology and data processing.

Additional file 2: Table S1. ASV abundance and taxonomy, includes the taxonomy of the samples used in this study, which was obtained after 16S rRNA sequencing.

Additional file 3: Table S2. Differentially abundant ASVs, contains the results of the differential abundance analysis for the samples Marine bacterial communities and Culturable communities

Additional file 4: Table S3. Bacterial isolates, taxonomy and antibiotic resistance, contain the collection of isolates, their culture media, antibiotic resistance results, taxonomy and 16S rRNA gene sequences.

Additional file 5: Table S4. Metagenome, ARG prediction and classification, contains the ARG prediction using CARD of the PSh and AFS metagenomes, their abundance and classification.

## Data Availability

The raw sequences used in this study are available in NCBI SRA repository, as part of the PRJNA971915 bioproject, under the accession IDs SRR24758006 and SRR24758005, corresponding to biosamples SAMN35521964 and SAMN35521965 for PSh and AFS, respectively. The sequences of the 16S rRNA genes were deposited in Genbank under the accession numbers given in Additional file 4.

## Abbreviations

AFS: Aquaculture farm site
PSh: Pacific shore
ARB: Antibiotic resistance bacteria
ARG: Antibiotic resistance gene
ARGs: Antibiotic resistance genes
ASV: Amplicon sequence variant
NM: Nutrient medium
SM: Saline medium
FFC: Florfenicol
FLQ: Flumequine
OTC: Oxytetracycline
PCR: Polymerase chain reaction
NM–FFC, –FLQ, –OTC: Nutrient medium supplemented with the antibiotics FFC, FLQ or OTC
SM–FFC, –FLQ, –OTC: Saline medium supplemented with the antibiotics FFC, FLQ or OTC
