# Supplementary material for: Impact of salmon farming in the antibiotic resistance and structure of marine bacterial communities from surface seawater of a northern Patagonian area of Chile
Source: Biol Res. 2024 Nov 10;57:84. doi: 10.1186/s40659-024-00556-4 (PMC11552226; doi:10.1186/s40659-024-00556-4)
Supplement: Supplementary file 1 — Additional file 1: Methods S1. Extended sample collection protocol, methodology and data processing. [file 40659_2024_556_MOESM1_ESM.pdf]

Additional file 1. Extended methodology and data processing.

Sample collection

Two sampling sites were chosen based on their proximity to farming sites: one from a salmon farm in the western side of the island of Chiloé, which is in a saturated area of aquaculture, and one site from the eastern side of the same island, near the protected area “Monumento natural Islotes de Puñihul”. The surface seawater from the salmon farm (Aquaculture Farm Site, AFS) and the Pacific Ocean surface seawater (Pacific shore site, PSh) were collected in November 2019 (spring time) in areas with approximately 30 m deep using sterile opaque plastic containers. The pH of the water at different sampling sites ranged from 7.9–8.2, salinity of ~44 mS and temperature of 11 °C. Temperature and conductivity profiles were obtained using a IP67 combo pH/ conductivity/ tds/ salinity/ d.o. meter model 86021. Measurements were taken concurrent with water sample collection, and are shown in the following table.

Location	Site	Coordinates	Date	Salinity	Dissolved oxygen	pH	Tempera ture
Puñihuil locality, Chiloé province, west Pacific shore, Chile	PSh	41°55'13.7"S 74°03'17.8"W	07/11/19	44.2 mS	8.80 mg/L	8.24	11.6 °C
Quicaví locality, Chiloé province, Chile.	AFS	42°17'06.7"S 73°14'22.1"W	06/11/19	43.9 mS	8.86 mg/L	7.92	11.0 °C

The map shown in Figure 1A was obtained from https://www.d-maps.com/carte.php?num_car=186348&lang=en and modified using the software Adobe Illustrator version 25.3.1.

Microbiological analyses of culturable microbiota

The microorganisms captured in the 0.22 µm Whatman filters were recovered using sterile cotton swabs, placed in 5 ml of sterile sea water and vortexed for 30 seconds 3 times. For each sample, serial dilutions (10^0, 10^{-1}, 10^{-2}, 10^{-3}, 10^{-4}) were plated in agar media supplemented with 0.25 µg/mL amphotericin B (Gibco, Thermo Fisher) to prevent fungal growth, in 7 different culture media: Instant Ocean salts agar (0.75% in distilled water with 15 g/L agar (Difco)), Seawater agar (sterile seawater obtained from each sampling site with 15 g/L agar (Difco)), R2A agar (Difco) in distilled water, R2A agar dissolved in sterile sea water, R2A agar with instant ocean salts (IO, 0.75% in distilled water), LB agar diluted 10 times in distilled water (1 g/L Peptone (Difco), 0.5 g/L Yeast Extract (Difco), 1 g/L NaCl (Merck), and 15 g/L agar (Difco)), and TSAYE agar (15 g/L Bacto Tryptone (Difco), 5 g/L Bacto Soytone (Difco), 6 g/L Yeast Extract (Difco), 5 g/L

Two sampling sites were chosen based on their proximity to farming sites: one from a salmon farm in the western side of the island of Chiloé, which is in a saturated area of aquaculture, and one site from the eastern side of the same island, near the protected area "Monumento natural Islotes de Puñihul". The surface seawater from the salmon farm (Aquaculture Farm Site, AFS) and the Pacific Ocean surface seawater (Pacific shore site, PSh) were collected in November 2019 (spring time) in areas with approximately 30 m deep using sterile opaque plastic containers. The pH of the water at different sampling sites ranged from 7.9–8.2, salinity of ~44 mS and temperature of 11 °C. Temperature and conductivity profiles were obtained using a IP67 combo pH/ conductivity/ tds/ salinity/ d.o. meter model 86021. Measurements were taken concurrent with water sample collection, and are shown in the following table.

Location	Site	Coordinates	Date	Salinity	Dissolved oxygen	pH	Tempera-ture
Puñihuil locality, Chiloé province, west Pacific shore, Chile	PSh	41°55'13.7"S 74°03'17.8"W	07/11/19	44.2 mS	8.80 mg/L	8.24	11.6 °C
Quicaví locality, Chiloé province, Chile.	AFS	42°17'06.7"S 73°14'22.1"W	06/11/19	43.9 mS	8.86 mg/L	7.92	11.0 °C

The map shown in Figure 1A was obtained from https://www.d-maps.com/carte.php?num_car=186348&lang=en and modified using the software Adobe Illustrator version 25.3.1.

Microbiological analyses of culturable microbiota

The microorganisms captured in the 0.22 µm Whatman filters were recovered using sterile cotton swabs, placed in 5 ml of sterile sea water and vortexed for 30 seconds 3 times. For each sample, serial dilutions (10^0, 10^{-1}, 10^{-2}, 10^{-3}, 10^{-4}) were plated in agar media supplemented with 0.25 µg/mL amphotericin B (Gibco, Thermo Fisher) to prevent fungal growth, in 7 different culture media: Instant Ocean salts agar (0.75% in distilled water with 15 g/L agar (Difco)), Seawater agar (sterile seawater obtained from each sampling site with 15 g/L agar (Difco)), R2A agar (Difco) in distilled water, R2A agar dissolved in sterile sea water, R2A agar with instant ocean salts (IO, 0.75% in distilled water), LB agar diluted 10 times in distilled water (1 g/L Peptone (Difco), 0.5 g/L Yeast Extract (Difco), 1 g/L NaCl (Merck), and 15 g/L agar (Difco)), and TSAYE agar (15 g/L Bacto Tryptone (Difco), 5 g/L Bacto Soytone (Difco), 6 g/L Yeast Extract (Difco), 5 g/L sodium chloride, 15 g/L agar (Difco)). Plates were incubated at 20-25 °C for 7 days and colonies were counted for each plate. Two additional media were tested, 0.75% Instant Ocean salts and

sterile Seawater with 15 g/L of agarose (Lafken) instead of agar, but after 14 days of incubation no bacterial growth was observed in both media.

Except for the Instant Ocean salts agar and the Seawater agar, colonies were observed at day 4 after incubation, so the remaining five media with fastest growing bacteria were selected to create the isolates collection (R2A in dH_2O, diluted LB 10%, R2A in seawater, R2A with IO salts and TSAYE). From each media, 20 colonies with visible macroscopic differences were recovered and isolated to generate a collection of approximately 100 colonies from each sampling site.

In addition, pools were created from the plates with observed bacterial growth after 7 days by scrapping all bacterial growth recovered from every dilution of each culture media. Bacteria were suspended in sterile PBS, homogenized by vortexing and used to create the "Culturable community" pool. This was created by combining equal amounts of bacteria from each culture media, thus obtaining 5 replicates per site of bacterial pools from 7 culture media. Aliquots of the Culturable communities were pelleted and stored at 4 °C until DNA purifications, and also to use as inoculum for the diluted LB agar ("Nutrient medium") and R2A agar in seawater ("Saline medium"), the two media selected based on their contrasting composition and diversity of colonies. After incubation at 20-25 °C for 7 days, bacteria were collected, re-suspended in sterile PBS, and use for subsequent antibiotic susceptibility assays. Antibiotics used by the Chilean salmon industry were selected for the susceptibility assays [1] in the Saline and Nutrient media, in the following concentrations: 10 µg/L florfenicol (FFC, Sigma-Aldrich), 10 µg/L flumequine (FLQ, Sigma-Aldrich), or 25 µg/L oxytetracycline (OTC, Sigma-Aldrich). Colonies were counted in the media with and without antibiotics to calculate the frequency of resistant colonies, as the ratio between the number of resistant colonies and the total number of colonies obtained in a particular medium without antibiotics. Bacteria grown in the Nutrient medium and Saline medium, with and without antibiotics were collected as before, pelleted and stored at 4 °C until DNA purifications.

For the isolate collection, antibiotic susceptibility assays were performed in the agar media selected for each isolate (Table S3), using the antibiotic concentrations mentioned above. Individual colonies from each isolate were picked and used as inoculum for plating in 96-well plates with and without antibiotics. Bacterial growth was evaluated daily for up to 7 days, and the result (growth / no growth) was recorded for each isolate in the media supplemented with FFC, FLQ or OTC, and without antibiotics.

DNA purification

DNA was purified according to the protocol described by Tillet et al. 2000 [2] with some modifications. The filters and the bacterial pellets were suspended in xanthogenate buffer [1% potassium ethyl xanthogenate (Sigma-Aldrich, United States), 100 mM TrisHCl (pH 7.4), 20 mM EDTA (pH 8), 800 mM ammonium acetate] with 1% SDS. The mixture was incubated at 65 °C

for 2 h and placed on ice for 30 min. The DNA was then purified with phenol-chloroform-isoamyl alcohol (25:24:1), followed by chloroform isoamyl alcohol (24:1). The DNA was precipitated overnight with cold isopropanol (-20 °C) and subsequently washed with 70% ethanol. The DNA quality was evaluated by spectrophotometry (A260/A280 ratio) in a Nanoquant spectrophotometer (Tecan), and the integrity was verified by standard 1% agarose gel electrophoresis. DNA was quantified using a Qubit 2.0 Fluorometer (Thermo Fisher Scientific).

16S rRNA gene amplicon sequencing and analysis

Bacterial samples from the Marine bacterial communities, Culturable communities, the Nutrient media (with and without antibiotics), and the Saline medium (with and without antibiotics), were collected in quintuplicate. Each DNA was purified and the V1–V3 hypervariable region of the 16S rRNA gene was amplified using the primer set 28F (5′-GA GTT TGA TCM TGG CTC AG-3′) and 519R (5′-GWA TTA CCG CGG CKG CTG-3′). Sequencing was performed by Mr. DNA (Shallowater, TX, USA) using an Illumina MiSeq platform in an overlapping 2×300bp configuration, with a minimum throughput of 20,000 reads per sample. Sequencing outputs were raw sequence data information. The Fastq processor application on the website www.mrdnafreesoftware.com was used to create the file formats required by QIIME2 for downstream analysis.

Bioinformatics analysis was performed using QIIME 2 2022.11 [3]. Raw sequences of the 16S rRNA gene were demultiplexed and quality filter using q2-demux plugin followed by denoising with DADA2 [4] (via q2-dada2). Taxonomy of the 16S rRNA sequences was assigned to amplicon sequence variants (ASVs) using the q2-feature-classifier classify-sklearn naïve Bayes [5] against Silva 138 99% database [6]. ASVs that were taxonomically annotated as “Mitochondria” and “Chloroplast” were excluded from the bacterial community analysis. To remove sparse ASVs only those ASVs present in at least three of the five replicates were used in the subsequent analyses (Table S1). All ASVs that have passed the filters were aligned using Mafft (via q2-alignment) [7] and used to construct a phylogeny using fasttree2 [8] (via q2-phylogeny). After rarefaction (subsampling without replacement) alpha-diversity metrics (Observed richness and Shannon index), and beta diversity metrics (Bray-Curtis dissimilarity) were estimated using q2-diversity core-metric. Alpha diversity was compared among the different groups of samples using Kruskal–Wallis test (“qiime diversity alpha-group-significance”) and p-values were corrected using a Benjamini and Hochberg correction method and false discovery rate (FDR) [9] as multiple comparisons method ($FDR < 0.05$ was considered statistically significant). For beta diversity, Principal Coordinate Analysis (PcoA) was performed using Bray-Curtis distance matrices and permutational (nonparametric) multivariate statistic (PERMANOVA, allowing for 999 permutations) was used to evaluate significance differences. Ward’s hierarchical clustering method based on Euclidean distance was also applied using

MicrobiomeAnalyst program [10] after filtering samples to remove 10% of low variance counts (based on inter-quantile range) and scaled by using Total sum scaling (TSS). Differentially abundant taxa from Marine bacterial communities and Culturable communities were identified by DESeq2 analysis [11], as shown in the diagram below. Briefly, this tool evaluates differential abundance of taxa, assuming negative binomial distribution for read counts. They compare null (common parameters) and alternative (distinct parameters) hypotheses for each taxon. Rejection of the null hypothesis indicates differential abundance. Test were perform using DEseq2 implemented in microbiomeMarker R package, v. 1.6 [12] (Table S2).

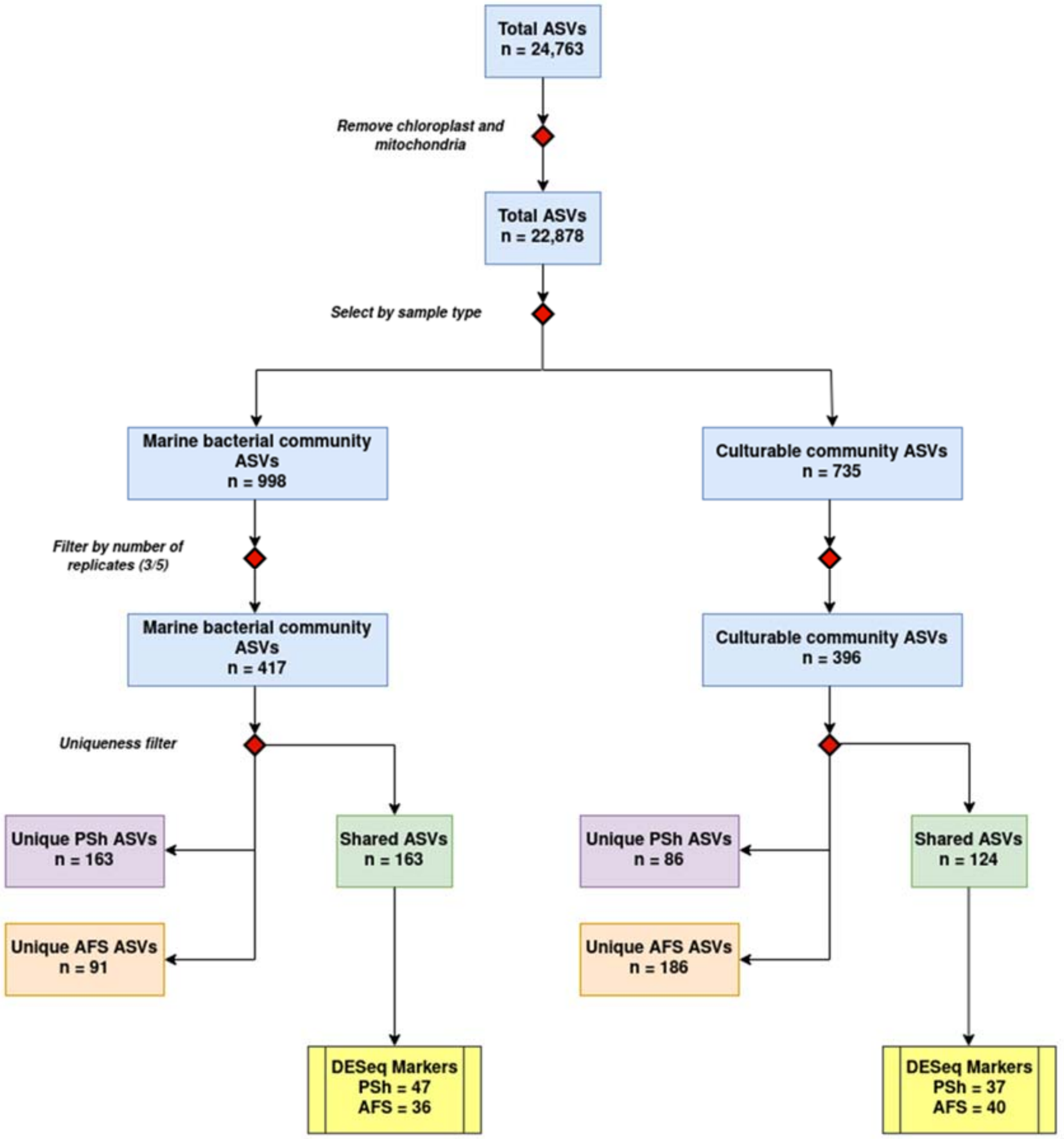

The amplicon sequence data are deposited in NCBI under BioProject ID PRJNA971915.

For the isolate collection, the genomic DNA purified from individual colonies was used as template to amplify the full-length 16S rRNA gene using the universal primers 27F (50-AGAGTTTGATCCTGGCTCAG-30) and 1492R (50-CGGTTACCTTGTTACGACTT-30) (Jiang et al., 2006). The reactions were carried out in 25 µL reactions using the GoTaq® Green

Master Mix (Promega, Madison, WI, USA), with 10 µM of each primer. The following PCR conditions were used: initial denaturation at 95 °C for 10 min, 30 cycles of 95 °C for 60 s, 60 °C for 30 s and 72 °C for 90 s, with a final extension at 72 °C for 10 min. The amplification products were visualized on 1% agarose gels by ethidium bromide staining and UV illumination. The PCR products from the 16S rRNA gene amplification were purified and sequenced in Macrogen (Seoul, Korea). Taxonomy was assigned using BLAST against both, NCBI16S RefSeq and RDP (Ribosomal Database Project, release 11) databases. Isolates taxonomy is listed in Table S3.

Library preparation and shotgun metagenomics sequencing

The DNA purified from PSh or AFS Marine bacterial community were pooled and used for metagenomics library construction. CD Genomics Services Company (NY, USA) performed shotgun sequencing on the Illumina NovaSeq platform, generating approximately 40 million paired-end reads of 150pb (equivalent to 12 Gb data) per sample. A total of 1 µg of DNA per sample was used as input material for DNA sample preparation. Sequencing libraries were generated using the NEBNext® Ultra™ DNA Library Prep Kit for Illumina (NEB, USA) following the manufacturer's recommendations. Briefly, the DNA samples were barcoding and then fragmented by sonication to a size of 350 bp, and then the DNA fragments were end-polished, A-tailed, and ligated with the full-length adapter for Illumina sequencing with further PCR amplification. Finally, PCR products were purified using the AMPure XP system, and libraries were analyzed for size distribution by Agilent2100 Bioanalyzer and quantified using real-time PCR. The qualified library was sequenced on an Illumina Novaseq6000.

Metagenome assemblies and analysis

The quality of the metagenome raw sequences was evaluated with the FastQC program [13]. Following identification of aspects with low quality scores, the Prinseq tool [14] was used to filter the reads based on several parameters, including trimming sequences at the 5' end from base 1 to base 15, filtering by a quality score of q=20 (allowing for 1 error every 100 base pairs), removal of reads less than 70 bp in length, and use of the Dust algorithm to classify and remove low complexity sequences. Additionally, identical sequences were removed as they were attributed to artifacts in the sequencing process using the *derep* function. Metagenome assemblies were performed using meta-SPADES [15] with default parameters and eliminated resulting contigs of less than 500 bp. The quality of the assemblies was evaluated using metaQUAST [16], and genes and proteins were predicted using Prodigal v2.6.3 in "meta" mode, ignoring the Shine-Dalgarno sequence [17] to allow prediction of genes and proteins from bacteria with different codon usage.

References

1. Miranda CD, Godoy FA, Lee MR. Current status of the use of antibiotics and the

antimicrobial resistance in the chilean salmon farms. Front Microbiol. 2018;9:1–14.

2. Tillett D, Neilan BA. Xanthogenate nucleic acid isolation from cultured and environmental cyanobacteria. J Phycol. 2000;36:251–8.

3. Bolyen E, Rideout JR, Dillon MR, Bokulich NA, Abnet CC, Al-Ghalith GA, et al. Reproducible, interactive, scalable and extensible microbiome data science using QIIME 2. Nat Biotechnol. 2019;37:852–7.

4. Callahan BJ, McMurdie PJ, Rosen MJ, Han AW, Johnson AJA, Holmes SP. DADA2: High-resolution sample inference from Illumina amplicon data. Nat Methods. 2016;13:581–3.

5. Bokulich NA, Kaehler BD, Rideout JR, Dillon M, Bolyen E, Knight R, et al. Optimizing taxonomic classification of marker-gene amplicon sequences with QIIME 2's q2-feature-classifier plugin. Microbiome. 2018;6:1–17.

6. Quast C, Pruesse E, Yilmaz P, Gerken J, Schweer T, Yarza P, et al. The SILVA ribosomal RNA gene database project: improved data processing and web-based tools. Nucleic Acids Res. 2013;41:D590-6.

7. Katoh K, Misawa K, Kuma KI, Miyata T. MAFFT: A novel method for rapid multiple sequence alignment based on fast Fourier transform. Nucleic Acids Res. 2002;30:3059–66.

8. Price MN, Dehal PS, Arkin AP. FastTree 2 - Approximately maximum-likelihood trees for large alignments. PLoS One. 2010;5.

9. Benjamini Y, Hochberg Y. Controlling the False Discovery Rate: A Practical and Powerful Approach to Multiple Testing. J R Stat Soc Ser B. 1995;57:289–300.

10. Dhariwal A, Chong J, Habib S, King IL, Agellon LB, Xia J. MicrobiomeAnalyst: A web-based tool for comprehensive statistical, visual and meta-analysis of microbiome data. Nucleic Acids Res. 2017;45:W180–8.

11. Love MI, Huber W, Anders S. Moderated estimation of fold change and dispersion for RNA-seq data with DESeq2. Genome Biol. 2014;15:1–21.

12. Cao Y, Dong Q, Wang D, Zhang P, Liu Y, Niu C. microbiomeMarker: an R/Bioconductor package for microbiome marker identification and visualization. Bioinformatics. 2022;38:4027–9.

13. Andrews S. FastQC: a quality control tool for high throughput sequence data. 2010. Available from: http://www.bioinformatics.babraham.ac.uk/projects/fastqc

14. Schmieder R, Edwards R. Quality control and preprocessing of metagenomic datasets. Bioinformatics. 2011;27:863–4.

15. Nurk S, Meleshko D, Korobeynikov A, Pevzner PA. MetaSPAdes: A new versatile metagenomic assembler. Genome Res. 2017;27:824–34.

16. Mikheenko A, Saveliev V, Gurevich A. MetaQUAST: Evaluation of metagenome assemblies. Bioinformatics. 2016;32:1088–90.

17. Hyatt D, Chen G-L, LoCascio PF, Land ML, Larimer FW, Hauser LJ. Prodigal: prokaryotic gene recognition and translation initiation site identification. BMC Bioinformatics. 2010;11:119.
